# Junk food diet-induced obesity increases D2 receptor autoinhibition in the ventral tegmental area and reduces ethanol drinking

**DOI:** 10.1371/journal.pone.0183685

**Published:** 2017-08-31

**Authors:** Jason B. Cook, Linzy M. Hendrickson, Grant M. Garwood, Kelsey M. Toungate, Christina V. Nania, Hitoshi Morikawa

**Affiliations:** 1 Waggoner Center for Alcohol and Addiction Research, The University of Texas at Austin, Austin, Texas, United States of America; 2 Division of Pharmacology and Toxicology, College of Pharmacy, The University of Texas at Austin, Austin, Texas, United States of America; 3 Department of Neuroscience The University of Texas at Austin, Austin, Texas, United States of America; University of Leicester, UNITED KINGDOM

## Abstract

Similar to drugs of abuse, the hedonic value of food is mediated, at least in part, by the mesostriatal dopamine (DA) system. Prolonged intake of either high calorie diets or drugs of abuse both lead to a blunting of the DA system. Most studies have focused on DAergic alterations in the striatum, but little is known about the effects of high calorie diets on ventral tegmental area (VTA) DA neurons. Since high calorie diets produce addictive-like DAergic adaptations, it is possible these diets may increase addiction susceptibility. However, high calorie diets consistently reduce psychostimulant intake and conditioned place preference in rodents. In contrast, high calorie diets can increase or decrease ethanol drinking, but it is not known how a junk food diet (cafeteria diet) affects ethanol drinking. In the current study, we administered a cafeteria diet consisting of bacon, potato chips, cheesecake, cookies, breakfast cereals, marshmallows, and chocolate candies to male Wistar rats for 3–4 weeks, producing an obese phenotype. Prior cafeteria diet feeding reduced homecage ethanol drinking over 2 weeks of testing, and transiently reduced sucrose and chow intake. Importantly, cafeteria diet had no effect on ethanol metabolism rate or blood ethanol concentrations following 2g/kg ethanol administration. In midbrain slices, we showed that cafeteria diet feeding enhances DA D2 receptor (D2R) autoinhibition in VTA DA neurons. These results show that junk food diet-induced obesity reduces ethanol drinking, and suggest that increased D2R autoinhibition in the VTA may contribute to deficits in DAergic signaling and reward hypofunction observed with obesity.

## Introduction

The reinforcing properties of addictive drugs and palatable foods are mediated, in part, by the mesostriatal dopamine (DA) system [1]. Moreover, extended exposure to drugs of abuse, including ethanol, or energy dense palatable foods produce similar DAergic neuroadaptations. For example, chronic exposure to ethanol and other drugs of abuse reduces D2 receptors (D2Rs) and basal DA levels in the striatum [2–4], which is also observed with energy dense food consumption [5–7]. Obese humans also have reduced D2R expression in the striatum [8] and reduced striatal activation in response to palatable food [9]. Therefore, since neuroadaptations following energy dense food or chronic drug exposure are similar, overconsumption of energy dense foods may increase drug addiction susceptibility. Interestingly, rodent studies have shown that high fat or sugar consumption reduces psychostimulant intake and conditioned place preference [10–13]. In contrast, prior high fat or sugar/carbohydrate consumption can increase [14, 15] or decrease [16, 17] ethanol drinking in rodents. However, it is not known how consumption of junk food items regularly consumed by humans affects ethanol drinking.

In the US approximately 35% of adults and 17% of children and adolescents are obese [18]. The rising prevalence of obesity has been associated with increased accessibility to “junk foods” high in fat, sugar, and other carbohydrates [19], and the consumption of these diets is especially prominent during adolescence [20–22]. In an attempt to model this type of energy dense diet contributing to obesity, investigators have given rats access to junk food items, termed a cafeteria diet [5, 6, 23]. Cafeteria diet feeding has been shown to reduce D2Rs and basal DA levels in the striatum, reduce sensitivity of reward circuitry using intracranial self-stimulation, and produce compulsive-like food consumption [5, 6]. However, it is not known if cafeteria diet feeding alters electrophysiological properties of DA neurons in the ventral tegmental area (VTA) or influences ethanol drinking.

Somatodendritic DA release activates D2Rs on the somata and dendrites of DA neurons resulting in autoinhibition *in vivo* [24, 25] and *in vitro* [26, 27] by activation of G protein-gated inwardly rectifying potassium channels (GIRK) via G_i/o_ signaling. Thus, D2R activation of GIRK results in hyperpolarization and reduced neuronal excitability [28]. In VTA DA neurons, repeated administration of ethanol or acute cocaine administration increases D2R-mediated autoinhibition [29, 30]. Furthermore, following repeated ethanol administration in mice, the increase in D2R autoinhibition was associated with increased homecage ethanol drinking [29]. Although it is clear high calorie diets produce addictive-like DAergic adaptations in the striatum, the effects of high calorie diets on D2R autoinhibition in VTA DA neurons has not been characterized.

In the current study, we investigated the effects of cafeteria diet on homecage ethanol or sucrose drinking, VTA DA neuron basal firing frequency, and D2R-mediated autoinhibition of VTA DA neurons. Cafeteria diet feeding during adolescence resulted in an obese-like phenotype and a long-lasting reduction in ethanol drinking using a 2 hr drinking in the dark (DID) ethanol presentation that produces moderate ethanol intake. Importantly, cafeteria diet feeding had no effect on blood ethanol concentrations (BECs) or ethanol metabolism rate following a 2 g/kg intraperitoneal (i.p.) ethanol injection. Furthermore, cafeteria diet feeding increased D2R-mediated autoinhibition of VTA DA neurons.

## Methods and materials

SubjectsMale Wistar rats were obtained from Harlan laboratories (Indianapolis, IN) at 3 weeks old. Rats were single housed in Plexiglass cages, which on one side of the cage had a Plexiglass platform measuring 7” x 4” x 1.25” secured to the floor for cafeteria diet placement. All rats had standard laboratory chow available *ad libitum* and water was available at all times except during ethanol or sucrose drinking sessions. The vivarium was maintained on a reverse 12 hr light-dark cycle (light onset at 0100 hr), constant temperature of 22 ± 2°C, and 65% relative humidity. Animal care and handling procedures followed National Institutes of Health Guidelines under The University of Texas at Austin Institutional Animal Care and Use Committee approved protocols.

### Cafeteria diet feeding

Once per day (1 hr into the dark cycle) a cafeteria diet consisting of high calorie junk food items including cheesecake (Atlanta Cheesecake Company, Kennesaw, GA), bacon (H-E-B, San Antonio, TX), cookies (Chips Ahoy/Oreo, Nabisco, East Hanover, NJ; sugar wafer, Vista, Sheare’s Foods, Massillon, OH), potato chips (Lays Classic/Ruffles, Frito Lay, Plano, TX) high sugar breakfast cereals (CoCo Puff, General Mills, Minneapolis, MN; Froot Loops, Kellog, Battle Creek, MI), marshmallows (Kraft, Northfield, IL), or chocolate candies (M&M, MARS, McLean, VA) was provided to the cafeteria diet group. Four of the cafeteria diet food items were administered per day and variety of diet was maintained by alternating food items daily. The chow only group received only laboratory chow (LabDiet,Prolab RMH 1800, St. Louis, MO), which was also available to the cafeteria diet group ad libitum. Macronutrient content (based on calories provided) of the chow only diet consisted of 14% fat, 65% carbohydrate, and 21% protein, and on average the cafeteria diet consisted of 42% fat, 52% carbohydrate, and 6% protein. Cafeteria diet was administered for 3 weeks for caloric intake and D2R outward current experiments (starting at approximately 3–4 weeks old) and for 4 weeks for all other experiments (starting at approximately 5 weeks old). For caloric intake measurements, cafeteria diet and chow only food was weighed daily and caloric intake was calculated using macronutrient information provided from the manufacturer.

### Homecage ethanol or sucrose drinking

One week after habituation, rats were given 2 hr/day limited access to an ethanol (10% v/v) or sucrose (5% w/v) solution to assess baseline drinking. During all ethanol or sucrose drinking sessions the homecage water bottle was replaced with a bottle containing the ethanol or sucrose solution at 1 hr into the dark cycle. Following baseline ethanol or sucrose drinking (7 days), rats were randomly assigned to the cafeteria diet or chow only group. Next, rats were fed cafeteria diet or chow only for 4 weeks. Twenty-four hours after the last cafeteria diet administration, rats began daily ethanol or sucrose drinking sessions.

### Blood ethanol concentration (BEC)

Following 4 weeks of cafeteria diet or chow only feeding, rats were administered ethanol (2g/kg, 15% v/v in saline, i.p.) 24 hr following the last cafeteria diet administration. Whole blood samples (10 μL) were collected via tail snip at 30, 60, and 120 min following ethanol injection and added to glass gas chromatography (GC) vials containing 90 μL of 5M sodium chloride. Sample ethanol concentrations were analyzed on the same day as blood collection with GC using a Bruker 430-GC (Bruker Corporation, Fremont, CA) equipped with a flame ionization detector and Combi PAL autosampler. Briefly, each sample was warmed to 65°C for 3 min before the solid-phase microextraction fiber (SPME; 75 μm CAR/PDMS, fused silica; Supelco) absorbed the ethanol vapor for 3 min. The SPME fiber then desorbed the sample into the GC injection port for 1 min at 220°C. Helium (8.5 mL/min flow rate) was used as the carrier gas and an HP Innowax capillary column (30 m x 0.53 mm x 1 μm film thickness; Agilent Technologies, Santa Clara, CA) was used for separation. External ethanol standards (25, 50, 100, 200, 400, and 600 mg/dL) were analyzed to calculate a standard curve. Chromatograms were analyzed using CompassCDS Workstation software (Bruker Corporation, Fremont, CA), and the peak heights for ethanol (~2 min retention time) were used to construct a standard curve and interpolate sample ethanol concentrations.

### Electrophysiology

Rats were anesthetized with isoflurane and the brain was removed and dissected in cold cutting solution containing (in mM) 205 sucrose, 2.5 KCl, 1.25 NaH_2_PO_4_, 7.5 MgCl_2_, 0.5 CaCl_2_, 10 glucose, and 25 NaHCO_3_, saturated with 95% O_2_, and 5% CO_2_ (~300mOsm/kg). Horizontal midbrain slices (200 μm) were sectioned on a vibratome and allowed to recover for 1 hr in artificial cerebrospinal fluid (aCSF) at 34°C. Recordings were performed in the lateral VTA 50–150 μm from the medial border of the medial terminal nucleus of the accessory optic tract. During recording, slices were perfused with oxygenated, warmed (34°C) aCSF (in mM) 126 NaCl, 2.5 KCl, 1.2 NaH_2_PO_4_, 1.2 MgCl_2_, 2.4 CaCl_2_, 11 glucose, 21.4 NaHCO_3_. Cell-attached loose-patch recordings (~20 MΩ seal) were performed with pipettes containing 150mM NaCl. Whole-cell recordings were performed with pipettes containing an intracellular solution consisting of (in mM) 115 K-methylsulfate or K-gluconate, 20 KCl, 1.5 MgCl_2_, 10 HEPES, 0.025 EGTA, 2 Mg-ATP, 0.2 Na_2_-GTP, and 10 Na_2_-phosphocreatine (pH 7.2–7.3, ~285 mOsm kg^-1^). Putative DA neurons were identified by their spontaneous low frequency pacemaker firing (1–5 Hz) and broad action potentials (> 1.2 ms) in cell-attached configuration, and large I_h_ (>200 pA) in response to a 1.5 sec voltage step from -62mV to -112 mV in whole-cell voltage-clamp mode. Voltage-clamp recordings were made at a holding potential of -62mV, corrected for a liquid junction potential of -7 mV. Whole-cell recordings were discarded if series resistance increased above 20 MΩ or input resistance dropped below 200 MΩ. Data was filtered at 1–5 kHz and digitized at 2–10 kHz.

### Data analysis

Data are expressed as mean ±SEM. Statistical significance was determined by Student’s t-test or two-way ANOVA followed by Bonferroni post hoc test.

## Results

### Cafeteria diet access results in high caloric intake and an obese-like phenotype

**Fig 1:**
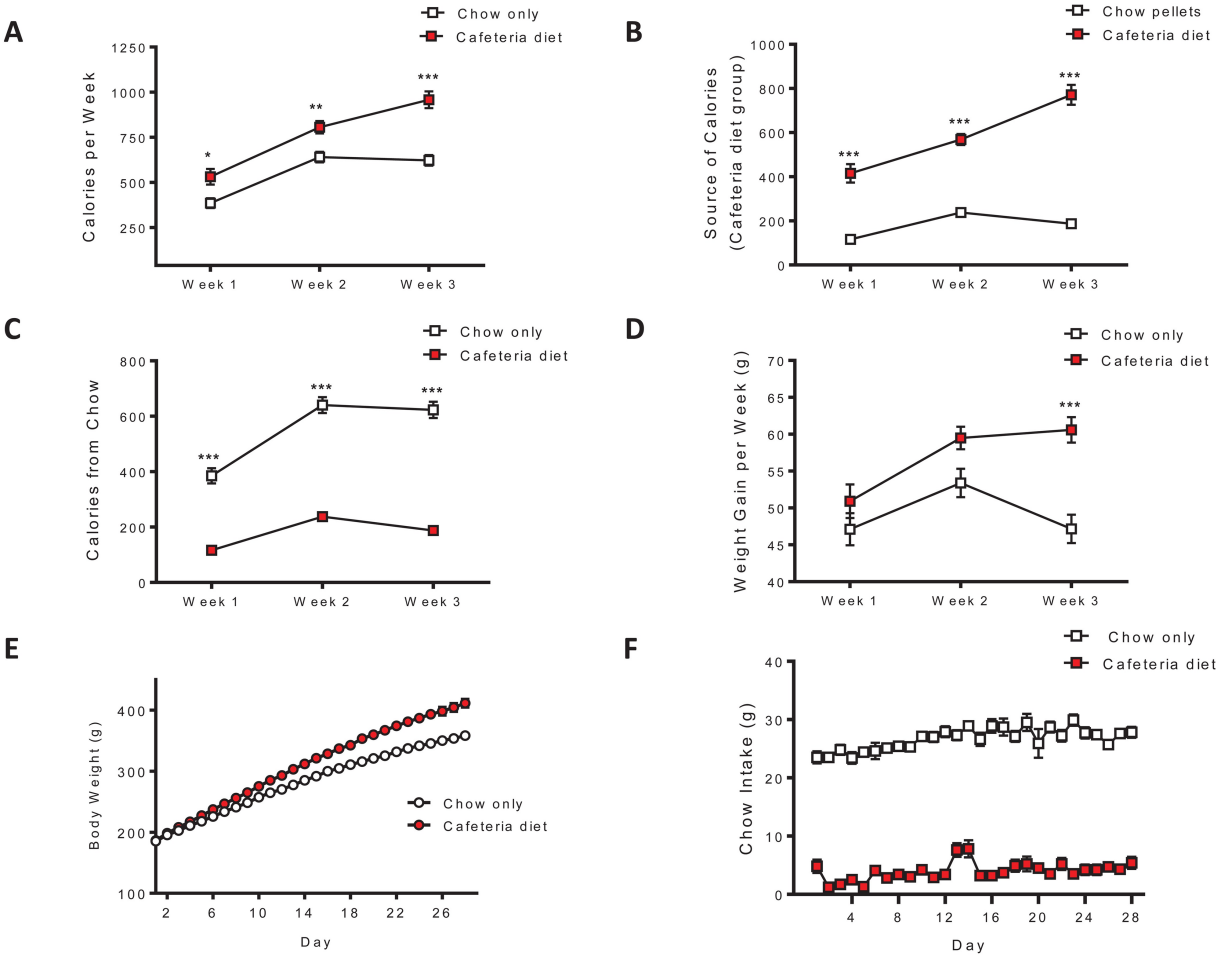
Cafeteria diet access results in elevated caloric intake and an obese-like phenotype. Caloric intake and the source of calories were assessed over 3 weeks. (A) Rats with daily access to cafeteria diet consumed significantly more calories over the 3 weeks of feeding than the chow only group (n = 14-19/group). (B) The cafeteria diet group consumed significantly more calories from cafeteria diet food items than from chow pellets (n = 19). (C) The chow only group consumed more calories from chow pellets than the cafeteria diet group (n = 14-19/group). (D) Cafeteria diet access resulted in increased weight gain over the 3 weeks of feeding (n = 14-19/group). (E) Four weeks of cafeteria diet feeding significantly increased body weight, compared to chow only fed controls (main effect of diet, p < 0.001, two-way ANOVA, n = 44-46/group). (F) Throughout the 4 weeks of cafeteria diet access, the cafeteria diet group consumes significantly less chow than the chow only group (main effect of diet, p < 0.0001, two-way ANOVA, n = 10-11/group). * p < 0.05, ** p < 0.01, *** p < 0.001, Bonferroni post hoc test.

Caloric intake for the cafeteria diet and chow only groups as well as the source of calories for the cafeteria diet group were assessed over 3 weeks. The cafeteria diet group consumed more calories than the chow only group over the 3 weeks of feeding (interaction: F_(2,62)_ = 22.43, p < 0.0001; diet: F_(1,62)_ = 17.41, p < 0.001; time F_(2,62)_ = 254.7, p < 0.0001; Fig 1A). The cafeteria diet group consumed significantly more calories from cafeteria diet food items than from chow pellets throughout the 3 weeks of feeding (interaction: F_(2,72)_ = 57.22, p < 0.0001; diet: F_(1,72)_ = 117.2, p < 0.0001; time F_(2,72)_ = 110.5, p < 0.0001; Fig 1B). Calories derived from chow pellets was significantly greater for the chow only group during the 3 week assessment (interaction: F_(2,62)_ = 28.80, p < 0.0001; diet: F_(1,62)_ = 196.3, p < 0.0001; time F_(2,62)_ = 150.0, p < 0.0001; Fig 1C). Ultimately, the cafeteria diet group displayed a greater degree of weight gain over the 3 weeks of feeding (interaction: F_(2,62)_ = 8.188, p < 0.001; diet: F_(1,62)_ = 10.62, p < 0.005; time F_(2,62)_ = 18.48, p < 0.0001; Fig 1D). Four weeks of cafeteria diet feeding resulted in an obese-like phenotype with body weights significantly heavier than the chow only group (interaction: F_(27,2376)_ = 44.48, p < 0.0001; diet: F_(1,2376)_ = 14.89, p < 0.001; time F_(27,2376)_ = 2634, p < 0.0001; Fig 1E). Furthermore, in a different group of animals we showed that the cafeteria diet group eats very little chow during the 4 weeks of cafeteria diet feeding compared to controls (interaction: F_(27,486)_ = 3.039, p < 0.0001; diet: F_(1,486)_ = 601.7, p < 0.0001; time F_(27,486)_ = 8.097, p < 0.0001; Fig 1F). These results show that cafeteria diet access resulted in overeating highly palatable junk foods and the subsequent loss of homeostatic energy balance.

### Prior cafeteria diet feeding reduced homecage ethanol drinking with no effect on ethanol metabolism rate or BECs

**Fig 2:**
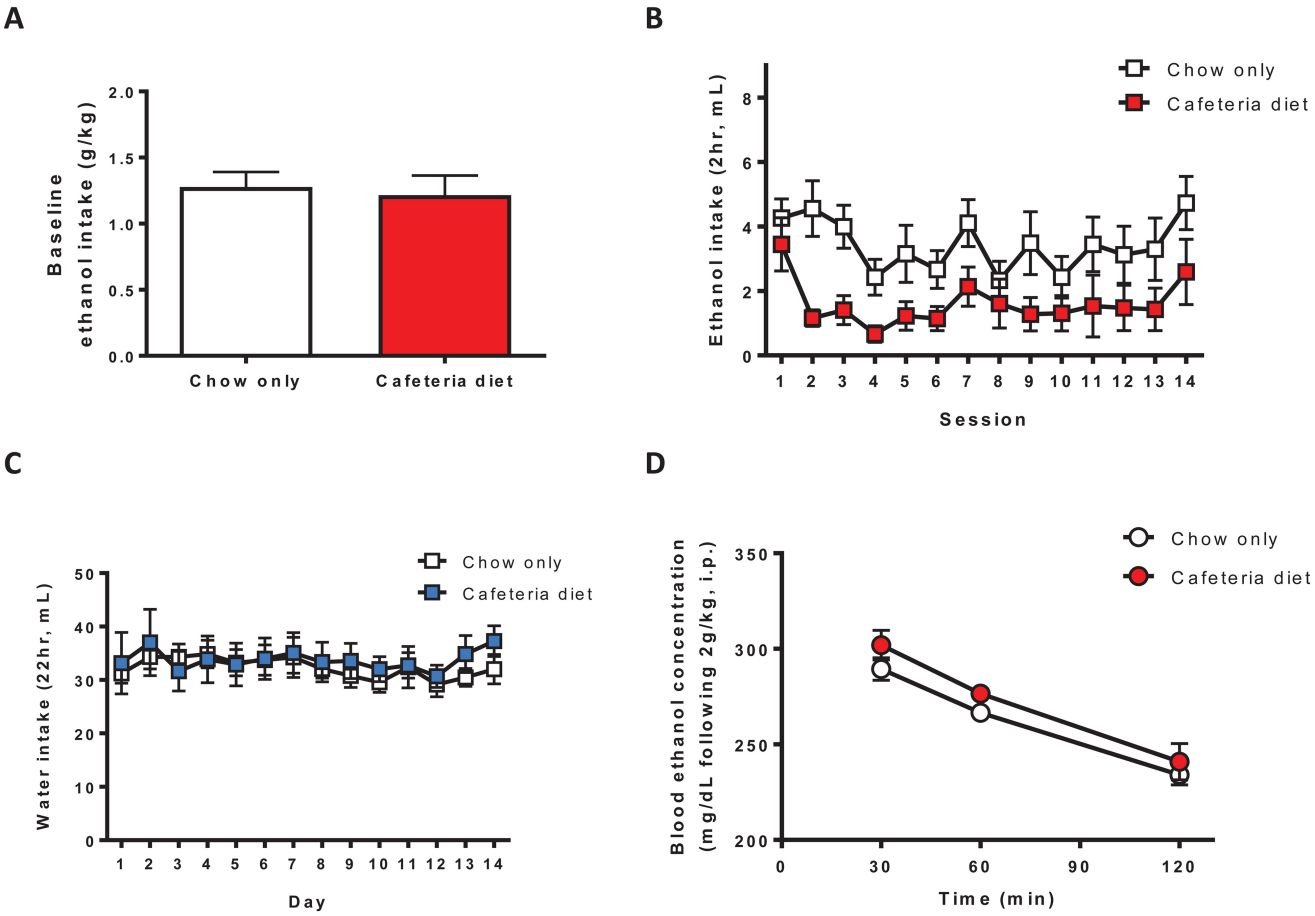
Prior cafeteria diet feeding reduces ethanol drinking with no effect on ethanol metabolism rate or BECs. (A) Mean baseline ethanol drinking (g/kg) over the 7 days prior to cafeteria diet feeding was similar between groups (p = 0.7480, Student’s t-test, n = 6-7/group). (B) Prior cafeteria diet feeding (4 weeks) reduced the total volume of ethanol (10%, v/v, 2hr/day) consumed during the 2 weeks of testing (main effect of diet, p < 0.05, two-way ANOVA, n = 6-7/group), (C) with no effect on total water consumption (n = 6-7/group). (D) There was no difference in the slopes of BECs (30–120 min following a 2g/kg administration, i.p.) between groups (p = 0.6535, linear regression, n = 4-5/group). BECs were similar between groups at 30, 60, and 120 min post-ethanol administration. BEC, blood ethanol concentration; i.p., intraperitoneal.

To determine the effects of prior cafeteria diet feeding on ethanol drinking we used a DID 2hr limited access homecage ethanol (one bottle, 10% v/v) drinking procedure that produces moderate levels of ethanol intake. Baseline ethanol drinking (g/kg) averaged over the 7 days prior to cafeteria diet access was similar between groups (t_(11)_ = 0.3295, p = 0.7480; Fig 2A). However, following 4 weeks of cafeteria diet feeding, the total volume of ethanol consumed was reduced during the 2 weeks of testing (diet: F_(1,143)_ = 5.635, p < 0.05; time F_(13, 143)_ = 3.638, p < 0.0001; Fig 2B). The magnitude of the reduction in ethanol drinking was larger when ethanol intake was plotted in g/kg since body weights for the cafeteria diet and chow only groups are markedly different. Ethanol intake in g/kg averaged over the 2 weeks of testing was 0.67 ± 0.11 g/kg for the chow only group and 0.25 ± 0.06 g/kg for the cafeteria diet group. Water consumption over 2 weeks of testing was not different between groups (diet: F_(1,143)_ = 0.1280, p = 0.7273; Fig 2C).

Since cafeteria diet feeding may change metabolic processes, including ethanol metabolism, we administered ethanol (2g/kg, i.p.) and measured BECs at 30, 60, and 120 min post-injection following cafeteria diet or chow only feeding. Using linear regression to compare BEC slopes (30–120 min post-injection) between groups, cafeteria diet feeding had no effect on ethanol metabolism rate (p = 0.6535; Fig 2D). Furthermore, there was no difference in BECs between groups (diet: F_(1,14)_ = 2.056). Therefore, changes in ethanol metabolism rate or ethanol absorption into the bloodstream cannot explain reduced ethanol drinking following cafeteria diet feeding.

### Prior cafeteria diet feeding transiently altered homecage sucrose drinking and chow intake

**Fig 3:**
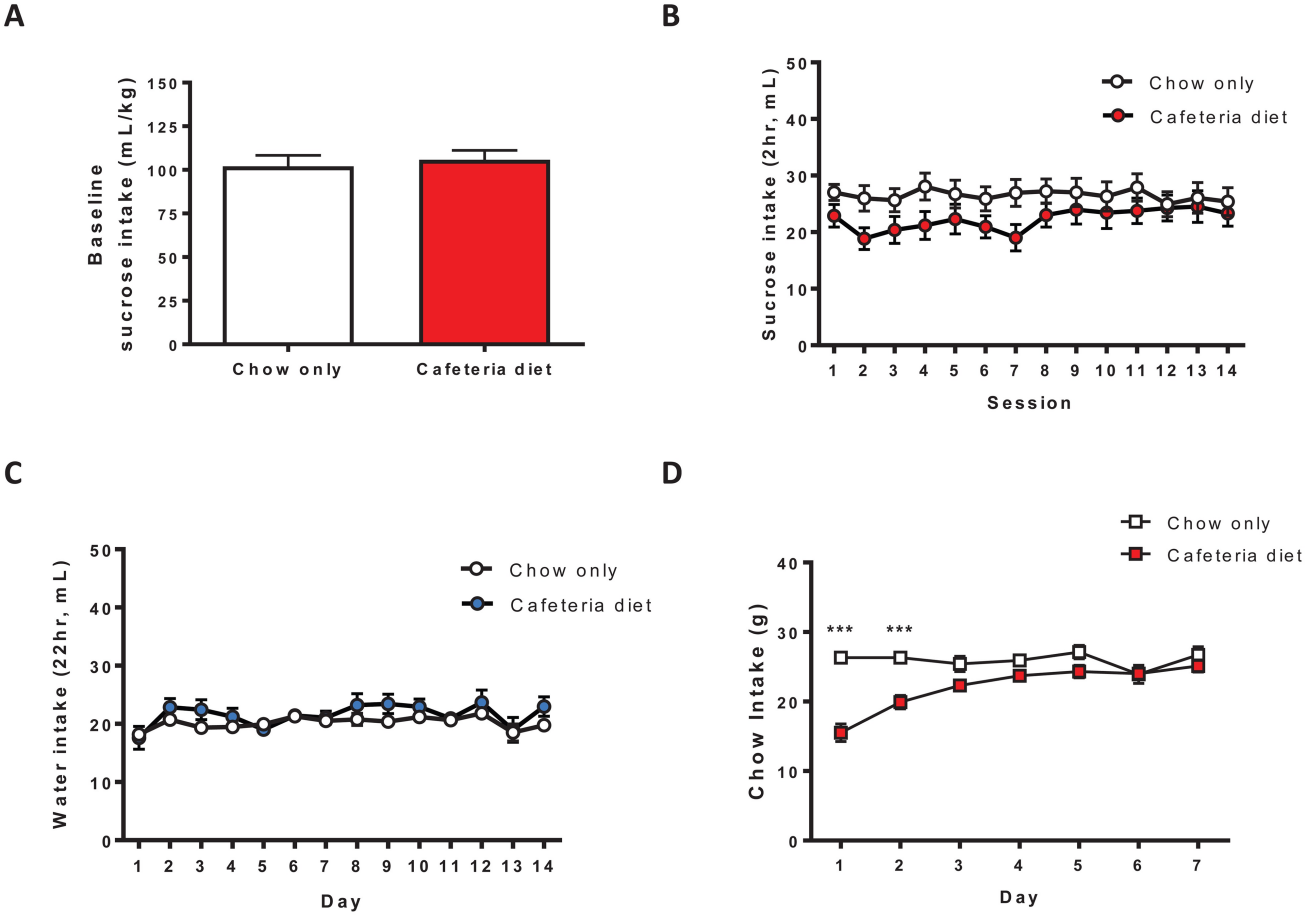
Prior cafeteria diet feeding transiently reduced sucrose drinking and chow intake. (A) Mean baseline sucrose drinking (mL/kg) over the 7 days prior to cafeteria diet feeding was similar between groups (p = 0.6489, Student’s t-test, n = 15-16/group). (B) Prior cafeteria diet feeding (4 weeks) transiently altered sucrose (5%, w/v, 2hr/day) consumption (diet x time interaction, p < 0.005, two-way ANOVA, n = 15-16/group). (C) There was no difference in water consumption between groups (n = 15-16/group). (D) Prior cafeteria diet feeding transiently reduced chow intake (n = 10/group). *** p < 0.001, Bonferroni post hoc test.

To determine if cafeteria diet alters consumption of other reinforcing solutions we tested the effects of prior cafeteria diet feeding on homecage sucrose drinking. Using a similar DID 2hr limited access sucrose (one bottle, 5% w/v) drinking procedure, baseline sucrose drinking (mL/kg) was similar between groups (t_(29)_ = 0.4600, p = 0.6489; Fig 3A). Prior cafeteria diet feeding transiently reduced sucrose drinking (diet x time interaction: F_(13,377)_ = 2.520, p < 0.005; Fig 3B). Although there was a significant diet x time interaction, post hoc analysis did not reach significance at any timepoint. However, based on the data (Fig 3B), the interaction between diet group and time can be explained by a transient reduction in sucrose drinking following cafeteria diet feeding. By the second week of testing, however, sucrose drinking was similar to the chow only group. Water consumption over 2 weeks of testing was not different between groups (diet: F_(1,377)_ = 1.176, p = 0.2870; Fig 3C). Similar to a previous study [31], cafeteria diet feeding transiently reduced chow intake (interaction: F_(6,110)_ = 12.46, p < 0.0001; diet: F_(1,110)_ = 15.46, p < 0.005; time F_(6,110)_ = 10.97, p < 0.0001; Fig 3D) for 2 days following cafeteria diet feeding (Bonferroni posthoc test, p < 0.001; Fig 3D). Therefore, cafeteria diet exposure produces a long-lasting reduction in ethanol drinking and transient reductions in sucrose drinking and chow intake.

### Cafeteria diet increases D2R autoinhibition in VTA DA neurons

**Fig 4:**
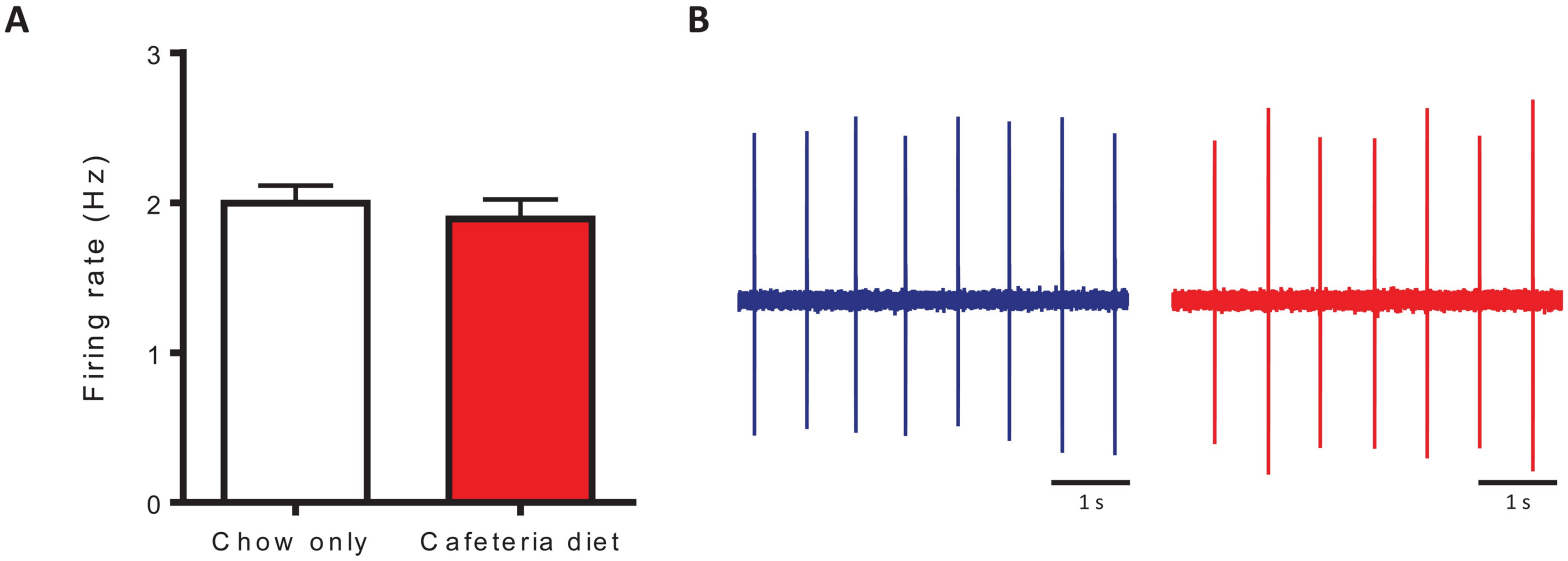
Cafeteria diet feeding had no effect on basal tonic pacemaker firing frequency of VTA DA neurons. (A) Basal tonic firing frequency of VTA DA neurons was similar between groups (p = 0.4681, Student’s t-test, n = 36-38/group). (B) Representative traces of VTA DA neuron firing following 4 weeks of chow only (blue) or cafeteria diet (red) feeding. DA, dopamine; VTA; ventral tegmental area.

**Fig 5:**
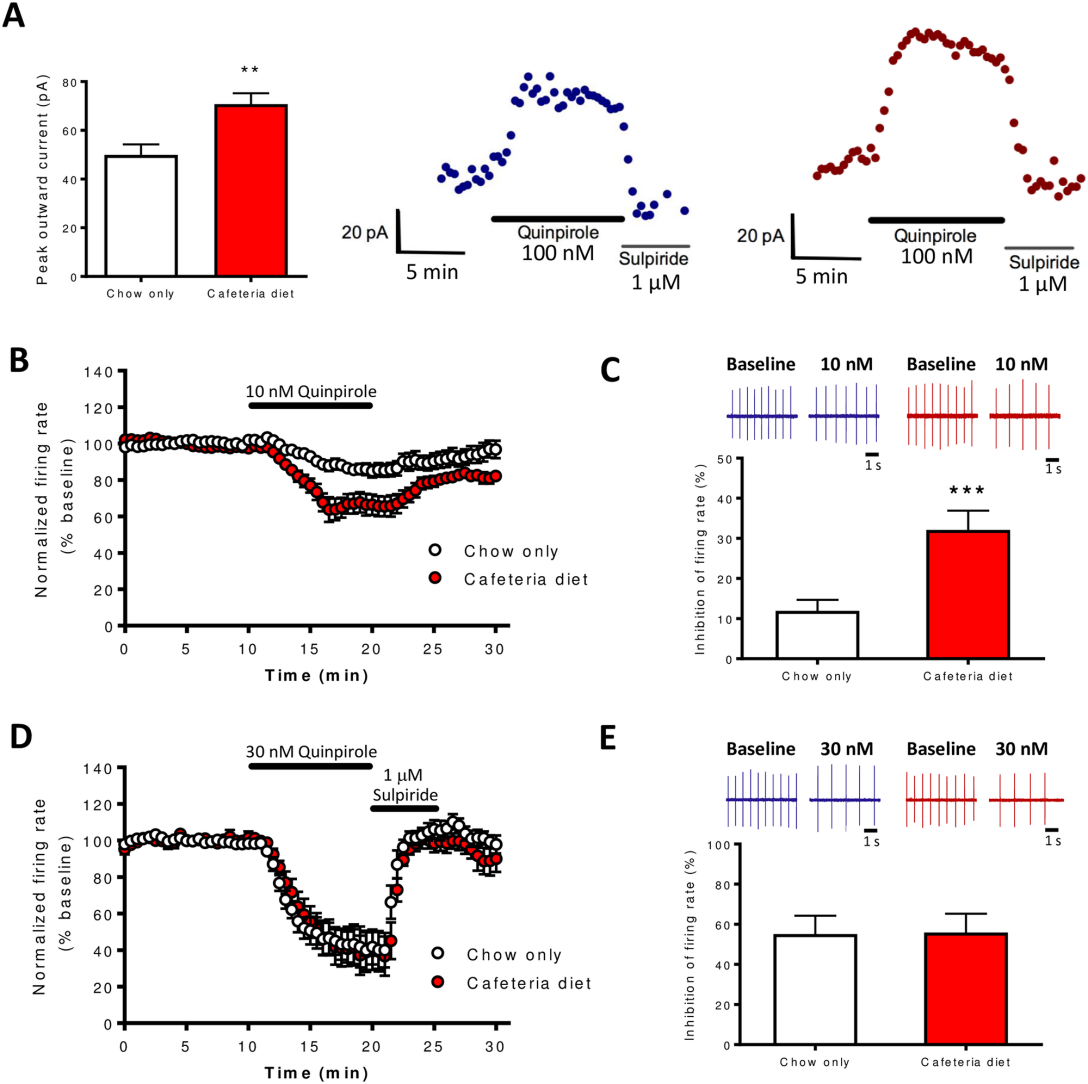
Cafeteria diet feeding increases D2R-mediated autoinhibition of VTA DA neurons. (A) Cafeteria diet increased the mean peak amplitude of quinpirole-mediated (100 nM) inhibitory outward GIRK currents compared to chow only controls. Quinpirole was bath applied for 10 min and sulpiride (1 μM) rapidly reversed the quinpirole-mediated current. Examples of quinpirole-mediated outward currents (V_h_ = -62 mV) for chow only (blue) or cafeteria diet fed (red) rats (n = 16-25/group). (B) Cafeteria diet feeding increased the inhibitory effects of 10 nM quinpirole on VTA DA neuron firing frequency over 10 min of quinpirole bath application (main effect of diet, p < 0.0005, two-way ANOVA, n = 13-16/group) and (C) quinpirole-mediated percent inhibition of firing frequency (p < 0.001, Student’s t-test). Representative traces of DA neuron firing frequency during baseline or 10 nM quinpirole application following chow only (blue) or cafeteria diet (red) feeding. (D-E) Inhibition of DA neuron firing frequency by 30 nM quinpirole was similar between groups (n = 9/group). Following 10 min of 30 nM quinpirole bath application, sulpiride (1 μM) was applied to the bath to rapidly reversed quinpirole-mediated inhibition of firing frequency. Representative traces of DA neuron firing frequency during baseline or 30 nM quinpirole application following chow only (blue) or cafeteria diet (red) feeding. *** p < 0.001, Student’s t-test. DA, dopamine; D2R, dopamine D2 receptor; GIRK, G protein-gated inwardly rectifying potassium channels; VTA; ventral tegmental area.

Brain slice electrophysiology was used to examine the effects of cafeteria diet on basal firing frequency and D2R-mediated autoinhibition of VTA DA neurons. Cafeteria diet feeding had no effect on basal tonic pacemaker firing frequency of VTA DA neurons (t_(72)_ = 0.7294, p = 0.4681; Fig 4A and 4B). Next, we tested the effects of cafeteria diet on D2R-mediated outward currents using the D2R agonist quinpirole. Cafeteria diet feeding increased the mean peak amplitude of quinpirole-mediated (100nM) inhibitory outward currents (t_(39)_ = 3.167, p < 0.005; Fig 5A) compared to controls. Furthermore, cafeteria diet increased the inhibitory effects of 10nM quinpirole on firing frequency of VTA DA neurons during 10 min of quinpirole administration (interaction: F_(19,513)_ = 5.425, p < 0.0001; diet: F_(1,513)_ = 16.40, p < 0.0005; time F_(19,513)_ = 39.24, p < 0.0001; Fig 5B), and percent inhibition of firing frequency produced by quinpirole (t_(27)_ = 3.824, p < 0.001; Fig 5C). At a higher concentration of quinpirole (30nM) there was no difference in the inhibition of firing frequency during 10 min of quinpirole administration (diet: F_(1,304)_ = 0.1049, p = 0.7502; Fig 5D) or percent inhibition of firing between groups (t_(16)_ = 0.05265, p = 0.9587; Fig 5E). Therefore, cafeteria diet exposure increased quinpirole-mediated (100nM) outward currents and increased the sensitivity of quinpirole-mediated inhibition of firing frequency.

## Discussion

The goal of the current study was to examine the effects of cafeteria diet feeding on homecage ethanol drinking and VTA DA neuron physiology. Prior cafeteria diet feeding reduced ethanol drinking during 2 weeks of testing, but had no effect on ethanol metabolism rate or BECs following a 2g/kg (i.p.) ethanol administration. It has been well documented that high calorie diets and diet-induced obesity lead to blunted DAergic signaling in the striatum, which has been postulated to contribute to deficits in reward [32–34]. However, the effects of diet-induced obesity on midbrain DA neurons have not been characterized. Here, we show that extended access to cafeteria diet increases D2R autoinhibition in VTA DA neurons, with no effect on basal tonic pacemaker firing frequency in the slice. It is not clear if increased D2R autoinhibition following cafeteria diet contributes to reduced ethanol drinking, however, increased DA neuron autoinhibition may contribute to reward hypofunction observed with obesity.

### Effects of cafeteria diet on ethanol drinking

Prior cafeteria diet feeding led to a prolonged reduction in homecage ethanol drinking. Total volume of ethanol consumed was reduced for 2 weeks following cafeteria diet exposure. Furthermore, cafeteria diet feeding had no effect on BECs or ethanol metabolism rate following 2g/kg ethanol administration. Therefore, reduced ethanol drinking cannot be explained by diet or body weight-induced changes in ethanol metabolism rate or ethanol absorption into the bloodstream. In contrast to ethanol drinking, sucrose drinking and chow intake were transiently reduced. Ultimately, prior cafeteria diet exposure produced a longer lasting reduction in ethanol intake, compared to natural reward intake.

It remains unclear how diet composition affects ethanol drinking in rodents. Therefore, in the current study, rats were fed a diet consisting of junk food items regularly consumed by humans. The current results are in agreement with a recent study showing that high fat diet-induced obesity or high fat diet-fed non-obese mice show reduced preference for ethanol [16]. Also, a high carbohydrate-low protein diet has been shown to reduce ethanol drinking in rats [17], however, ethanol intake was measured during diet exposure. Therefore, ethanol drinking may have been reduced based on caloric need instead of a reduction in the reinforcing properties of ethanol. In contrast, prior intermittent sucrose consumption (21 days) or a high fat diet (7 days) have been shown to increase homecage ethanol drinking [14, 15]. Both of these studies used 12 hr presentation of 4–5 increasing concentrations of ethanol (1,2,4,7, or 9%) for 4 days each, which is very different from the 2 hr access to 10% ethanol used in the present study. Discrepancies in the effects of high calorie diets on ethanol drinking could be due to differences in the nutrient content of the diet, duration and timing of the diet exposure, the ethanol drinking paradigm used, or strain/species specific effects.

### Effects of cafeteria diet on D2R autoinhibition

Cafeteria diet feeding increases D2R autoinhibition, which is also observed following repeated ethanol administration. Our lab has previously shown that repeated administration of ethanol in mice increases the potency of D2R-mediated outward currents in the VTA, and reduces a Ca^2+^ dependent desensitization of these currents [29]. In that study, repeated ethanol exposure increased the inhibitory effects of quinpirole on firing frequency at both 10 nM and 30 nM concentrations. However, cafeteria diet exposure increased the inhibitory effect of quinpirole on firing frequency only at the 10 nM concentration (Fig 5B and 5C). Although we did not determine if the potency/efficacy of quinpirole was altered by cafeteria diet, these results suggest that cafeteria diet increased the sensitivity of quinpirole to inhibit DA neuron firing. Acute cocaine administration (20 mg/kg) has also been shown to increase D2R-mediated outward currents in the substantia nigra pars compacta of mice [30]. In contrast, methamphetamine self-administration has been shown to reduce D2R-mediated currents in the VTA, which was also Ca^2+^ dependent [35]. Therefore, in contrast to neuroadaptations in the striatum where exposure to drugs of abuse or high calorie diets generally reduce D2R expression, specific drugs of abuse have divergent effects on D2R/GIRK-mediated currents. It should be noted that food restriction increases drug intake [36], including ethanol [37], and decreases D2R autoinhibition [38]. Since cafeteria diet exposure increases D2R autoinhibition and reduces ethanol intake, it will be important to determine the relationship between food intake, changes in D2R autoinhibition, and ethanol drinking. To our knowledge, there is only one other study that examined the effects of diet-induced obesity on D2R autoinhibition. In that study, high fat diet-induced obesity did not alter the inhibitory effects of a single dose of quinpirole (3–100 nM) on VTA DA neuron firing rate in mice [39]. However, stepwise application of quinpirole (3, 10, 30, and 100 nM) resulted in reduced inhibitory effects of quinpirole on firing, leading the authors to suggest that obese mice displayed accelerated D2R desensitization compared to control lean mice. It is not clear what underlies these discrepancies in the effects of cafeteria diet in rats versus high fat diet in mice on D2R autoinhibition. Further studies are warranted to determine the effects of energy dense diets and diet-induced obesity on VTA DA neurons and D2R autoinhibition.

It is not clear whether reduced ethanol drinking or the electrophysiological results were influenced by increased body weight in the current study. However, high calorie diets can dampen the DA system [13] and reduce ethanol drinking [16] in the absence of obesity. Increased adiposity is associated with changes in leptin, insulin, and ghrelin, all of which can modulate activity of the DA system [40–42]. Therefore, we cannot rule out that changes in homeostatic feeding mechanisms may have influenced the results. We also cannot rule out the possibility that cafeteria diet feeding may have altered circadian patterns of ingestive behavior since ethanol and sucrose drinking was only measured during a 2 hr access period.

The current study differs from previous studies [5, 6] that have examined the effects of cafeteria diet on the DA system by providing cafeteria diet feeding during adolescence instead of adulthood. Taken together, the data suggests that both adolescent and adult cafeteria diet feeding produce neuroadaptations that dampen the DA system and contribute to reward hypofunction. Although it is not known how cafeteria diet feeding during adulthood affects D2R autoinhibition, administration of drugs of abuse can increase D2R autoinhibition when administered during adolescence [29] or during adulthood [30].

### Significance of increased D2R autoinhibition and a hypodopaminergic state following cafeteria diet on ethanol drinking and consummatory behavior

*In vivo*, increased D2R autoinhibition may reduce basal DA neuron firing frequency, thus, dampening the DA system and contributing to a hypodopaminergic state. In the current study and a previous study from our lab [29], we did not detect basal DAergic tone in the slice as sulpiride fails to alter DA neuron firing frequency. However, *in vivo* the activity of DA neurons is continuously influenced by local DA and D2R autoinhibition. Therefore, increased D2R autoinhibition following cafeteria diet should lead to reduced basal firing rate of DA neurons in the intact animal, and contribute to a hypodopaminergic state thought to drive excessive food intake [19]. Similarly, extensive evidence from preclinical and human studies has led to the hypothesis that a hypodopaminergic state contributes to compulsive ethanol intake and relapse [43, 44]. The current results add to the growing literature suggesting cafeteria diet feeding produces addictive-like DAergic changes consistent with a hypodopaminergic state [5, 6]. Although a hypodopaminergic state has long been hypothesized to contribute to excessive ethanol drinking, cafeteria diet-induced hypodopaminergia does not translate into increased ethanol drinking. Previous work in our lab showed that repeated ethanol administration increased D2R autoinhibition, which was associated with increased homecage ethanol drinking in mice [29]. In that study, we concluded that increased D2R autoinhibition following repeated ethanol administration contributed to the hypodopaminergic state commonly observed with chronic ethanol exposure. Taken together, it seems plausible that expression of a hypodopaminergic state produced by chronic consumption of energy dense junk food leads to excessive/compulsive consummatory behavior that is reinforcer specific. Indeed, overconsumption of energy dense foods do not typically translate into excessive consumption of drugs of abuse, but instead, typically reduce drug intake. Previous evidence and the current results support this since extended access to cafeteria diet produces compulsive-like intake of palatable food [6], but reduces ethanol and sucrose drinking as we show here. Furthermore, chow intake was also transiently reduced following cafeteria diet feeding. Moreover, a high fat diet or sugar administration have been shown to reduce psychostimulant intake and conditioned place preference in rats [10–13]. Several large epidemiological studies also show that human obesity is generally not associated with alcohol or substance use disorders [45–48]. In contrast, there are limited studies providing evidence that previous high fat diet or sucrose administration increases ethanol drinking in rats [14, 15] or that obesity is associated with alcohol use disorders in humans [49]. Chronic ethanol administration may also produce reinforcer specific effects on drug consumption. For example, prior chronic ethanol exposure increases ethanol self-administration [50], but has no effect on cocaine self-administration [51], even though chronic administration of ethanol or cocaine both produce similar adaptations in the DA system. Furthermore, in terms of how a hypodopaminergic state affects ethanol drinking and relapse, a recent study examined changes in the DA system throughout the addiction cycle in rats and humans, and showed that abstinence is characterized by early hypodopaminergia followed by hyperdopaminergia during protracted abstinence, both of which may contribute to relapse vulnerability [52]. Therefore, deviations in DAergic signaling are associated with ethanol consumption and alcohol use disorders, but the precise relationship between DA signaling and ethanol drinking or relapse remains unclear.

### Significance of increased D2R autoinhibition in diet-induced obesity

Increased D2R autoinhibition may contribute to deficits in striatal DA transmission and reward hypofunction observed with diet-induced obesity. Obesity is associated with deficits in reward as well as motivational and emotional impairments often attributed in part to reduced DA signaling in the striatum [32, 33, 53]. Cafeteria diet feeding has been shown to reduce basal DA levels as well as levels of the DA metabolites 3,4-dihydroxyphenylacetic acid (DOPAC) and homovanillic acid (HVA) in the NAc [5]. Moreover, another study showed that obesity prone rats displayed 50% less basal DA in the NAc compared to controls [54]. These two studies provide evidence that cafeteria diet fed and obesity prone rats have presynaptic deficits in DA release using coronal NAc slice preparations. For example, obesity prone rats had reductions in the DA biosynthetic enzyme tyrosine hydroxylase and vesicular monoamine transporter 2 (VMAT2) that may reduce DA synthesis and release [54]. However, an overlooked aspect in the field is the involvement of physiological processes in the VTA where many of these DA neurons originate. The current results suggest that increased D2R autoinhibition may contribute to DAergic deficits observed with diet-induced obesity. Therefore, studies examining presynaptic mesolimbic mechanisms in the NAc and VTA may provide insight into neurobiological mechanisms contributing to obesity.

### Conclusions

Reduced DA transmission in the striatum of obese humans and rats have been well documented [6, 8, 19]. The present study suggests that increased D2R autoinhibition in the VTA may also contribute to diet-induced DA signaling deficits and reward hypofunction observed with obesity. Although high calorie diets and drugs of abuse produce similar changes in the mesolimbic DA system, we show that cafeteria diet feeding reduces ethanol drinking in rats. Overall, it appears that cafeteria diet-induced addictive-like changes in the DA system may specifically drive cafeteria diet consumption [6], while withdrawal from cafeteria diet results in prolonged suppression of ethanol drinking and transiently suppresses consumption of natural rewards (i.e., sucrose and chow pellets). These findings add to the growing literature showing that diet-induced obesity and drug addiction produce similar neuroadaptations in reward circuitry. Further investigation into midbrain DAergic adaptations following excessive energy dense food or drug intake may lead to important insights into the mechanisms contributing to these major public health problems.

## Supporting information

S1 Data Raw data for Fig 1. (XLSX)

S2 Data Raw data for Fig 2. (XLSX)

S3 Data Raw data for Fig 3. (XLSX)

S4 Data Raw data for Fig 4. (XLSX)

S5 Data Raw data for Fig 5. (XLSX)

## References

[1] Volkow ND, Wang GJ, Fowler JS, Tomasi D, Baler R. Food and drug reward: overlapping circuits in human obesity and addiction. Curr Top Behav Neurosci. 2012;11:1–24. Epub 2011/10/22. doi: 10.1007/7854_2011_169 . 22016109 10.1007/7854_2011_169

[2] Volkow ND, Wang GJ, Fowler JS, Logan J, Hitzemann R, Ding YS, et al Decreases in dopamine receptors but not in dopamine transporters in alcoholics. Alcohol Clin Exp Res. 1996;20(9):1594–8. Epub 1996/12/01. . 8986209 10.1111/j.1530-0277.1996.tb05936.x

[3] Moore RJ, Vinsant SL, Nader MA, Porrino LJ, Friedman DP. Effect of cocaine self-administration on dopamine D2 receptors in rhesus monkeys. Synapse. 1998;30(1):88–96. Epub 1998/08/15. doi: 10.1002/(SICI)1098-2396(199809)30:1<88::AID-SYN11>3.0.CO;2-L . 9704885 10.1002/(SICI)1098-2396(199809)30:1<88::AID-SYN11>3.0.CO;2-L

[4] Rossetti ZL, Hmaidan Y, Gessa GL. Marked inhibition of mesolimbic dopamine release: a common feature of ethanol, morphine, cocaine and amphetamine abstinence in rats. Eur J Pharmacol. 1992;221(2–3):227–34. Epub 1992/10/20. . 1426002 10.1016/0014-2999(92)90706-a

[5] Geiger BM, Haburcak M, Avena NM, Moyer MC, Hoebel BG, Pothos EN. Deficits of mesolimbic dopamine neurotransmission in rat dietary obesity. Neuroscience. 2009;159(4):1193–9. Epub 2009/05/05. doi: 10.1016/j.neuroscience.2009.02.007 ; 19409204 10.1016/j.neuroscience.2009.02.007 PMC2677693

[6] Johnson PM, Kenny PJ. Dopamine D2 receptors in addiction-like reward dysfunction and compulsive eating in obese rats. Nat Neurosci. 2010;13(5):635–41. Epub 2010/03/30. doi: 10.1038/nn.2519 ; 20348917 10.1038/nn.2519 PMC2947358

[7] Rada P, Bocarsly ME, Barson JR, Hoebel BG, Leibowitz SF. Reduced accumbens dopamine in Sprague-Dawley rats prone to overeating a fat-rich diet. Physiol Behav. 2010;101(3):394–400. Epub 2010/07/21. doi: 10.1016/j.physbeh.2010.07.005 ; 20643155 10.1016/j.physbeh.2010.07.005 PMC2930885

[8] Wang GJ, Volkow ND, Logan J, Pappas NR, Wong CT, Zhu W, et al Brain dopamine and obesity. Lancet. 2001;357(9253):354–7. Epub 2001/02/24. . 11210998 10.1016/s0140-6736(00)03643-6

[9] Stice E, Spoor S, Bohon C, Small DM. Relation between obesity and blunted striatal response to food is moderated by TaqIA A1 allele. Science. 2008;322(5900):449–52. Epub 2008/10/18. doi: 10.1126/science.1161550 ; 18927395 10.1126/science.1161550 PMC2681095

[10] Wellman PJ, Nation JR, Davis KW. Impairment of acquisition of cocaine self-administration in rats maintained on a high-fat diet. Pharmacol Biochem Behav. 2007;88(1):89–93. Epub 2007/09/04. doi: 10.1016/j.pbb.2007.07.008 ; 17764729 10.1016/j.pbb.2007.07.008 PMC2094387

[11] Kanarek RB, Mathes WF, Przypek J. Intake of dietary sucrose or fat reduces amphetamine drinking in rats. Pharmacol Biochem Behav. 1996;54(4):719–23. Epub 1996/08/01. . 8853195 10.1016/0091-3057(96)00012-3

[12] Davis JF, Tracy AL, Schurdak JD, Tschop MH, Lipton JW, Clegg DJ, et al Exposure to elevated levels of dietary fat attenuates psychostimulant reward and mesolimbic dopamine turnover in the rat. Behav Neurosci. 2008;122(6):1257–63. Epub 2008/12/03. doi: 10.1037/a0013111 ; 19045945 10.1037/a0013111 PMC2597276

[13] Hryhorczuk C, Florea M, Rodaros D, Poirier I, Daneault C, Des Rosiers C, et al Dampened Mesolimbic Dopamine Function and Signaling by Saturated but not Monounsaturated Dietary Lipids. Neuropsychopharmacology. 2016;41(3):811–21. Epub 2015/07/15. doi: 10.1038/npp.2015.207 ; 26171719 10.1038/npp.2015.207 PMC4707827

[14] Avena NM, Carrillo CA, Needham L, Leibowitz SF, Hoebel BG. Sugar-dependent rats show enhanced intake of unsweetened ethanol. Alcohol. 2004;34(2–3):203–9. Epub 2005/05/21. . 15902914 10.1016/j.alcohol.2004.09.006

[15] Carrillo CA, Leibowitz SF, Karatayev O, Hoebel BG. A high-fat meal or injection of lipids stimulates ethanol intake. Alcohol. 2004;34(2–3):197–202. Epub 2005/05/21. . 15902913 10.1016/j.alcohol.2004.08.009

[16] Takase K, Tsuneoka Y, Oda S, Kuroda M, Funato H. High-fat diet feeding alters olfactory-, social-, and reward-related behaviors of mice independent of obesity. Obesity (Silver Spring). 2016;24(4):886–94. Epub 2016/02/19. doi: 10.1002/oby.21441 . 26890672 10.1002/oby.21441

[17] Pekkanen L, Eriksson K, Sihvonen ML. Dietarily-induced changes in voluntary ethanol consumption and ethanol metabolism in the rat. Br J Nutr. 1978;40(1):103–13. Epub 1978/07/01. . 666993 10.1079/bjn19780100

[18] Ogden CL, Carroll MD, Kit BK, Flegal KM. Prevalence of childhood and adult obesity in the United States, 2011–2012. JAMA. 2014;311(8):806–14. Epub 2014/02/27. doi: 10.1001/jama.2014.732 . 24570244 10.1001/jama.2014.732 PMC4770258

[19] Volkow ND, Wise RA. How can drug addiction help us understand obesity? Nat Neurosci. 2005;8(5):555–60. Epub 2005/04/28. doi: 10.1038/nn1452 . 15856062 10.1038/nn1452

[20] Wang YC, Bleich SN, Gortmaker SL. Increasing caloric contribution from sugar-sweetened beverages and 100% fruit juices among US children and adolescents, 1988–2004. Pediatrics. 2008;121(6):e1604–14. Epub 2008/06/04. doi: 10.1542/peds.2007-2834 . 18519465 10.1542/peds.2007-2834

[21] Lustig RH, Schmidt LA, Brindis CD. Public health: The toxic truth about sugar. Nature. 2012;482(7383):27–9. Epub 2012/02/03. doi: 10.1038/482027a . 22297952 10.1038/482027a

[22] Vikraman S, Fryar CD, Ogden CL. Caloric Intake From Fast Food Among Children and Adolescents in the United States, 2011–2012. NCHS Data Brief. 2015;(213):1–8. . 26375457

[23] Heyne A, Kiesselbach C, Sahun I, McDonald J, Gaiffi M, Dierssen M, et al An animal model of compulsive food-taking behaviour. Addict Biol. 2009;14(4):373–83. Epub 2009/09/11. doi: 10.1111/j.1369-1600.2009.00175.x . 19740365 10.1111/j.1369-1600.2009.00175.x

[24] Pucak ML, Grace AA. Evidence that systemically administered dopamine antagonists activate dopamine neuron firing primarily by blockade of somatodendritic autoreceptors. J Pharmacol Exp Ther. 1994;271(3):1181–92. Epub 1994/12/01. . 7996424

[25] White FJ, Wang RY. A10 dopamine neurons: role of autoreceptors in determining firing rate and sensitivity to dopamine agonists. Life Sci. 1984;34(12):1161–70. Epub 1984/03/19. . 6708722 10.1016/0024-3205(84)90088-2

[26] Lacey MG, Mercuri NB, North RA. Dopamine acts on D2 receptors to increase potassium conductance in neurones of the rat substantia nigra zona compacta. J Physiol. 1987;392:397–416. Epub 1987/11/01. ; 2451725 10.1113/jphysiol.1987.sp016787 PMC1192311

[27] Beckstead MJ, Grandy DK, Wickman K, Williams JT. Vesicular dopamine release elicits an inhibitory postsynaptic current in midbrain dopamine neurons. Neuron. 2004;42(6):939–46. Epub 2004/06/23. doi: 10.1016/j.neuron.2004.05.019 . 15207238 10.1016/j.neuron.2004.05.019

[28] Luscher C, Slesinger PA. Emerging roles for G protein-gated inwardly rectifying potassium (GIRK) channels in health and disease. Nat Rev Neurosci. 2010;11(5):301–15. Epub 2010/04/15. doi: 10.1038/nrn2834 ; 20389305 10.1038/nrn2834 PMC3052907

[29] Perra S, Clements MA, Bernier BE, Morikawa H. In vivo ethanol experience increases D(2) autoinhibition in the ventral tegmental area. Neuropsychopharmacology. 2011;36(5):993–1002. Epub 2011/01/21. doi: 10.1038/npp.2010.237 ; 21248720 10.1038/npp.2010.237 PMC3077268

[30] Gantz SC, Robinson BG, Buck DC, Bunzow JR, Neve RL, Williams JT, et al Distinct regulation of dopamine D2S and D2L autoreceptor signaling by calcium. Elife. 2015;4 Epub 2015/08/27. doi: 10.7554/eLife.09358 ; 26308580 10.7554/eLife.09358 PMC4575989

[31] Rolls BJ, Rowe EA, Turner RC. Persistent obesity in rats following a period of consumption of a mixed, high energy diet. J Physiol. 1980;298:415–27. Epub 1980/01/01. ; 6987379 10.1113/jphysiol.1980.sp013091 PMC1279126

[32] Wang GJ, Volkow ND, Fowler JS. The role of dopamine in motivation for food in humans: implications for obesity. Expert Opin Ther Targets. 2002;6(5):601–9. Epub 2002/10/22. doi: 10.1517/14728222.6.5.601 . 12387683 10.1517/14728222.6.5.601

[33] Davis C, Strachan S, Berkson M. Sensitivity to reward: implications for overeating and overweight. Appetite. 2004;42(2):131–8. Epub 2004/03/11. doi: 10.1016/j.appet.2003.07.004 . 15010176 10.1016/j.appet.2003.07.004

[34] Blum K, Thanos PK, Gold MS. Dopamine and glucose, obesity, and reward deficiency syndrome. Front Psychol. 2014;5:919 Epub 2014/10/04. doi: 10.3389/fpsyg.2014.00919 ; 25278909 10.3389/fpsyg.2014.00919 PMC4166230

[35] Sharpe AL, Varela E, Bettinger L, Beckstead MJ. Methamphetamine self-administration in mice decreases GIRK channel-mediated currents in midbrain dopamine neurons. Int J Neuropsychopharmacol. 2015;18(5). Epub 2014/12/19. doi: 10.1093/ijnp/pyu073 ; 25522412 10.1093/ijnp/pyu073 PMC4376542

[36] Carroll ME, France CP, Meisch RA. Food deprivation increases oral and intravenous drug intake in rats. Science. 1979;205(4403):319–21. Epub 1979/07/20. 36665 10.1126/science.36665

[37] Middaugh LD, Kelley BM, Bandy AL, McGroarty KK. Ethanol consumption by C57BL/6 mice: influence of gender and procedural variables. Alcohol. 1999;17(3):175–83. Epub 1999/05/07. . 10231165 10.1016/s0741-8329(98)00055-x

[38] Branch SY, Goertz RB, Sharpe AL, Pierce J, Roy S, Ko D, et al Food restriction increases glutamate receptor-mediated burst firing of dopamine neurons. J Neurosci. 2013;33(34):13861–72. Epub 2013/08/24. doi: 10.1523/JNEUROSCI.5099-12.2013 ; 23966705 10.1523/JNEUROSCI.5099-12.2013 PMC3755722

[39] Koyama S, Mori M, Kanamaru S, Sazawa T, Miyazaki A, Terai H, et al Obesity attenuates D2 autoreceptor-mediated inhibition of putative ventral tegmental area dopaminergic neurons. Physiol Rep. 2014;2(5):e12004 Epub 2014/05/06. doi: 10.14814/phy2.12004 ; 24793981 10.14814/phy2.12004 PMC4098733

[40] Fulton S, Pissios P, Manchon RP, Stiles L, Frank L, Pothos EN, et al Leptin regulation of the mesoaccumbens dopamine pathway. Neuron. 2006;51(6):811–22. Epub 2006/09/20. doi: 10.1016/j.neuron.2006.09.006 . 16982425 10.1016/j.neuron.2006.09.006

[41] Labouebe G, Liu S, Dias C, Zou H, Wong JC, Karunakaran S, et al Insulin induces long-term depression of ventral tegmental area dopamine neurons via endocannabinoids. Nat Neurosci. 2013;16(3):300–8. Epub 2013/01/29. doi: 10.1038/nn.3321 ; 23354329 10.1038/nn.3321 PMC4072656

[42] Abizaid A, Liu ZW, Andrews ZB, Shanabrough M, Borok E, Elsworth JD, et al Ghrelin modulates the activity and synaptic input organization of midbrain dopamine neurons while promoting appetite. J Clin Invest. 2006;116(12):3229–39. Epub 2006/10/25. doi: 10.1172/JCI29867 ; 17060947 10.1172/JCI29867 PMC1618869

[43] Koob GF, Volkow ND. Neurocircuitry of addiction. Neuropsychopharmacology. 2010;35(1):217–38. Epub 2009/08/28. doi: 10.1038/npp.2009.110 ; 19710631 10.1038/npp.2009.110 PMC2805560

[44] Diana M. The dopamine hypothesis of drug addiction and its potential therapeutic value. Front Psychiatry. 2011;2:64 Epub 2011/12/07. doi: 10.3389/fpsyt.2011.00064 ; 22144966 10.3389/fpsyt.2011.00064 PMC3225760

[45] Pickering RP, Grant BF, Chou SP, Compton WM. Are overweight, obesity, and extreme obesity associated with psychopathology? Results from the national epidemiologic survey on alcohol and related conditions. J Clin Psychiatry. 2007;68(7):998–1009. Epub 2007/08/10. . 17685734 10.4088/jcp.v68n0704

[46] Simon GE, Von Korff M, Saunders K, Miglioretti DL, Crane PK, van Belle G, et al Association between obesity and psychiatric disorders in the US adult population. Arch Gen Psychiatry. 2006;63(7):824–30. Epub 2006/07/05. 63/7/824 doi: 10.1001/archpsyc.63.7.824 ; 16818872 10.1001/archpsyc.63.7.824 PMC1913935

[47] Scott KM, McGee MA, Wells JE, Oakley Browne MA. Obesity and mental disorders in the adult general population. J Psychosom Res. 2008;64(1):97–105. Epub 2007/12/26. doi: 10.1016/j.jpsychores.2007.09.006 . 18158005 10.1016/j.jpsychores.2007.09.006

[48] Scott KM, Bruffaerts R, Simon GE, Alonso J, Angermeyer M, de Girolamo G, et al Obesity and mental disorders in the general population: results from the world mental health surveys. Int J Obes (Lond). 2008;32(1):192–200. Epub 2007/08/23. doi: 10.1038/sj.ijo.0803701 ; 17712309 10.1038/sj.ijo.0803701 PMC2736857

[49] Barry D, Petry NM. Associations between body mass index and substance use disorders differ by gender: results from the National Epidemiologic Survey on Alcohol and Related Conditions. Addict Behav. 2009;34(1):51–60. Epub 2008/09/30. doi: 10.1016/j.addbeh.2008.08.008 ; 18819756 10.1016/j.addbeh.2008.08.008 PMC2645714

[50] Roberts AJ, Heyser CJ, Cole M, Griffin P, Koob GF. Excessive ethanol drinking following a history of ethanol dependence: animal model of allostasis. Neuropsychopharmacology. 2000;22:581–94. doi: 10.1016/S0893-133X(99)00167-0 10788758 10.1016/S0893-133X(99)00167-0

[51] Fredriksson I, Adhikary S, Steensland P, Vendruscolo LF, Bonci A, Shaham Y, et al Prior Exposure to Alcohol Has No Effect on Cocaine Self-Administration and Relapse in Rats: Evidence from a Rat Model that Does Not Support the Gateway Hypothesis. Neuropsychopharmacology. 2016 Epub 2016/09/22. doi: 10.1038/npp.2016.209 . 27649640 10.1038/npp.2016.209 PMC5506787

[52] Hirth N, Meinhardt MW, Noori HR, Salgado H, Torres-Ramirez O, Uhrig S, et al Convergent evidence from alcohol-dependent humans and rats for a hyperdopaminergic state in protracted abstinence. Proc Natl Acad Sci U S A. 2016;113(11):3024–9. Epub 2016/02/24. doi: 10.1073/pnas.1506012113 ; 26903621 10.1073/pnas.1506012113 PMC4801269

[53] Blum K, Liu Y, Shriner R, Gold MS. Reward circuitry dopaminergic activation regulates food and drug craving behavior. Curr Pharm Des. 2011;17(12):1158–67. Epub 2011/04/16. . 21492092 10.2174/138161211795656819

[54] Geiger BM, Behr GG, Frank LE, Caldera-Siu AD, Beinfeld MC, Kokkotou EG, et al Evidence for defective mesolimbic dopamine exocytosis in obesity-prone rats. Faseb J. 2008;22(8):2740–6. Epub 2008/05/15. doi: 10.1096/fj.08-110759 ; 18477764 10.1096/fj.08-110759 PMC2728544

